# Endoscopic stricturotomy in the treatment of anastomotic strictures in inflammatory bowel disease (IBD) and non-IBD patients

**DOI:** 10.1093/gastro/goz051

**Published:** 2019-10-21

**Authors:** Long-Juan Zhang, Nan Lan, Xian-Rui Wu, Bo Shen

**Affiliations:** 1 Laboratory of General Surgery, the First Affiliated Hospital, Sun Yat-Sen University, Guangzhou, Guangdong, P.R. China; 2 Center for Inflammatory Bowel Disease, Digestive Disease and Surgery Institute, The Cleveland Clinic Foundation, Cleveland, OH, USA; 3 Department of Colorectal Surgery and Guangdong Provincial Key Laboratory of Colorectal and Pelvic Floor Diseases, the Sixth Affiliated Hospital, Sun Yat-Sen University, Guangzhou, Guangdong, P.R. China

**Keywords:** endoscopic stricturotomy, inflammatory bowel disease, anastomotic stricture

## Abstract

**Backgrounds:**

Endoscopic stricturotomy (ESt) has been shown to be effective in treating inflammatory bowel disease (IBD)-associated anastomotic strictures. However, the outcome of ESt in benign, non-IBD conditions has not been described. The aim of this study was to evaluate the outcome of ESt in the management of IBD and non-IBD-associated strictures.

**Methods:**

Data of all consecutive IBD and non-IBD patients with benign anastomotic strictures treated with ESt from 2009 to 2016 were extracted. The primary outcomes were surgery-free survival and procedure-related complications.

**Results:**

A total of 49 IBD and 15 non-IBD patients were included in this study. The IBD group included 25 patients with Crohn’s disease and 24 with ulcerative colitis and ileal pouches. Underlying diseases in the non-IBD group included colorectal cancer (*n *=* *7), diverticulitis (*n *=* *5), large bowel prolapse (*n *=* *2), and constipation (*n *=* *1). Immediate technical success was achieved in all patients in both groups. Bleeding complications occurred on five occasions (4.7% per procedure) in the IBD group, while no complication occurred in the non-IBD group (*P *=* *0.20). Stricture improvement on follow-up endoscopy was found in 10 (20.4%) and 5 (33.3%) patients in the IBD and non-IBD groups, respectively (*P *=* *0.32). Six (12.2%) patients in the IBD group and four (26.7%) patients in the non-IBD group eventually required stricture-related surgery (*P *=* *0.23). IBD patients appeared to have a higher tendency for maintaining surgery-free after the procedure than non-IBD patients (*P *=* *0.08).

**Conclusions:**

Endoscopic stricturotomy was shown to have comparable outcomes, though non-IBD patients seem to have a higher need for subsequent surgery but a lower complication rate than IBD patients.

## Introduction

Strictures of the gastrointestinal (GI) tract are classified into primary (disease-related) and secondary types (surgical anastomosis-related) [1]. Etiological factors for the secondary anastomotic strictures include surgery-related ischemia, anastomotic dehiscence, pelvic infection, post-operative radiation, and the use of stapling devices [2, 3]. Anastomotic strictures resulting from fibrosis of the intestinal wall and the lacking of definitive anti-fibrotic drugs in inflammatory bowel disease (IBD) and non-IBD patients have made their management a difficult task [4, 5]. These strictures were traditionally treated with surgical stricturoplasty or additional surgical resection, but the surgical procedures can be technically challenging and the recurrence rate is high [6, 7]. Various endoscopic approaches such as endoscopic balloon dilation (EBD), endoscopic needle-knife stricturotomy (NKSt) or insulated-tip knife stricturotomy, stent placement, and local injection with long-acting corticosteroids have emerged as valid alternatives [8–12].

Endoscopic stricturotomy (ESt) is increasingly being performed for strictures of all types along the GI tract, including IBD and non-IBD-related benign primary or secondary/anastomotic strictures [8, 10, 13]. ESt in IBD patients was shown to be effective in spacing out the need for subsequent surgery with minimal post-procedural complications [8]. However, the efficacy and safety of ESt in benign, non-IBD anastomotic strictures have not been studied. Herein, the aim of this historical cohort was to evaluate the outcome of ESt in the management of benign anastomotic strictures in non-IBD patients in comparison to IBD patients.

## Patients and methods

### Data sources

All consecutive patients with a benign anastomotic stricture treated in our Interventional Inflammatory Bowel Disease (*i*-IBD) Unit at the Cleveland Clinic from 2009 to 2016 were identified. Demographic, clinical, and endoscopic features together with the management and outcomes were carefully reviewed. This study was approved by the Cleveland Clinic Institutional Review Board (IRB). Informed consent was obtained for the procedures. The clinical data of all patients with strictures who were treated with endoscopy in the *i*-IBD Unit were documented in the IRB-approved registry.

### Inclusion and exclusion criteria

The inclusion criteria were as follows: (i) patients with a benign anastomotic stricture and (ii) the stricture was only treated with ESt with or without prior, concurrent, or subsequent EBD. The exclusion criteria were as follows: (i) patients with the non-anastomotic, primary strictures; (ii) stricture suspicious of being malignant at the time of the ESt procedure; (ii) patients treated with endoscopic treatments other than EBD and ESt; or (iv) those with lack of follow-up.

### Data collection

General background information, such as age, gender, ethnicity, height, and weight, were extracted from the database. Height and weight were used to calculate the body mass index of each patient. Clinical histories were also extracted from the data along with previous surgical history and medication history. Current history of smoking was defined as consumption of more than seven cigarettes per week for at least 6 months and ex-smoker was defined as cessation of smoking at least 6 months prior to data entry. Significant comorbidities included were defined as previously established [14]. Recorded family history of IBD or colorectal cancer was those in the first-degree relatives. Indications of initial bowel surgery and post-operative neo-bowel anastomosis configuration were recorded in all patients.

The diagnosis of stricture was obtained by either endoscopy and/or abdominal imaging. Patients with stricture may or may not have stricture-related clinical symptoms. The degrees of the strictures were classified into traversable and non-traversable to the pediatric colonoscope. The length of stricture was measured based on endoscopy reports. Multiple strictures were defined as more than one stricture. Locations of strictures were classified as being ileocolonic anastomosis (ICA), ileorectal anastomosis (IRA), colo-colonic anastomosis, colo-rectal anastomosis (CRA), rectal-anal anastomosis, or ileal-pouch anal anastomosis (IPAA). Pre-procedural use of medications was defined as medications used within 1 month prior to the inception ESt procedure. The use of non-steroidal anti-inflammatory drug (NSAIDs) refers to the patients with NSAIDs as prescription medications as documented in the electronic medical records. It has been difficult to explore the use of over-the-counter medications, such as NSAIDs, in the retrospective approach. However, in our standard practice, we routinely discourage patients for the use of any NSAID at the time of IBD diagnosis, as a part of health-care education.

### Technique of endoscopic stricturotomy

**Figure 1.**
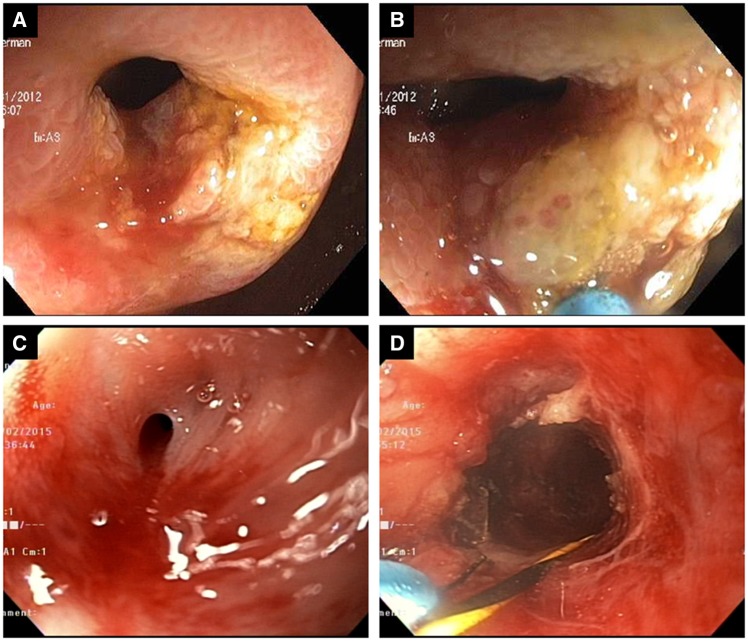
Endoscopic electroincision with a needle knife in the treatment of ileocolonic anastomotic stricture. (A) Stricture in Crohn’s disease patients at ileocolonic anastomosis. (B) Needle knife in action. (C) Stricture in non-inflammatory bowel disease patients at colorectal anastomosis. (D) Needle knife in action.

All ESt procedures were performed by an experienced endoscopist (B.S.). The stricture was treated with either a Boston Scientific triple-lumen needle knife (Boston Scientific, Marlborough, MA) or later on an Olympus single-use electrosurgical IT knife 2 (Olympus Medical Systems, Tokyo, Japan) under the setting of endoscopic retrograde cholangiopancreatography (ERCP) Endocut (ERBE USA Incorporated Surgical Systems, Marietta, GA). Strictures were incised in a circumferential or radial manner until adequate passage of the scope was achieved (Figure 1 and Supplementary Video).

### Outcome measurements

The primary outcomes were immediate technical success, defined as the passage of the pediatric colonoscope without resistance, and surgery-free survival. Patients who underwent subsequent surgery for their refractory stricture were evaluated in detail. Follow-up time was defined as the time from the first or inception ESt to the latest GI clinical/telephone follow-up or stricture-related surgery, whichever came first.

The secondary outcomes were procedure-related complications, endoscopic improvement, symptomatic improvement, as well as the disease-related hospitalization and emergency-department visits. Endoscopic improvement was defined as a shorter or more patent stricture in the immediate follow-up endoscope. Symptomatic improvement was determined by the comparison of the patient’s subjective symptoms at the follow-up visit immediately before and after the ESt. Hospital admission for planned surgery was not included as disease-related hospitalizations for analysis purposes. Planned surgery in this study was referring to patients who came back for planned diverting ostomy reversal. This meant that the patients were getting better and were ready to re-establish their bowel continuity. On the other hand, patients with procedure-associated complications who had salvage surgery were not counted towards planned surgery. The timing and modality of additional treatment that patients underwent before and after the initial ESt were documented. The interval between the inception ESt and subsequent endoscopic therapies (EBD and/or ESt) was calculated and compared.

### Statistical analysis

Descriptive statistics were computed for all variables. Categorical variables were summarized as percentages. Quantitative variables with a normal distribution were summarized as mean* *±* *standard deviation. Quantitative variables with paranormal distribution were summarized in median and interquartile range (IQR). Tests for the association between groups and categorical variables were performed using the chi-square test or Fisher’s exact test. For quantitative variables, the means were compared by Student’s *t*-test or Wilcoxon rank-sum test. The surgery-free survival was constructed using a Kaplan–Meier curve. A *P *<* *0.05 was considered as being statistically significant. All analyses were performed using SPSS software version 20.0 (SPSS, Chicago, IL).

## Results

### Demographics and clinical characteristics

A total of 64 patients with benign anastomotic strictures were involved in this study, including 49 IBD and 15 non-IBD patients. Patients in the IBD group were younger at the age of diagnosis for their primary disease (21.9 ± 8.5 vs 51.1 ± 14.8 years, *P *<* *0.01) as well as the age of the first surgical resection (30.0 ± 11.8 vs 55.1 + 13.3 years, *P *<* *0.01) than those in the non-IBD group. The duration from the diagnosis of primary disease to surgical bowel resection was longer in IBD patients than in non-IBD patients (3.2 [IQR: 0.04–13.2] vs 0.4 [IQR: 0.05–1.7] years, *P *<* *0.01) (Table 1).

**Table 1.** Characteristics of patients with anastomotic stricture

Characteristic	IBD patients (*n* = 49)	Non-IBD patients (*n* = 15)	*P*-value
Age at diagnosis for primary disease, years	21.9 ± 8.5	51.1 ± 14.8	<0.01
Age at the first surgery, years	30.0 ± 11.8	55.1 ± 13.3	<0.01
Duration from diagnosis to surgery, years	3.2 (0.04–13.2)	0.4 (0.05–1.7)	<0.01
Female	31 (63.3)	9 (60.0)	0.82
Baseline weight at ESt, kg	73.4 ± 16.6	75.1 ± 19.6	0.74
Baseline body mass index at ESt, kg/m^2^	26.3 (21.4–32.5)	23.0 (20.9–27.0)	0.48
History of smoking			0.21
Current	3 (6.1)	1 (6.7)	
Ex-smoker	9 (18.4)	6 (40.0)	
Never	37 (75.5)	8 (53.3)	
History of significant comorbidities*	4 (8.2)	6 (40.0)	0.01
History of autoimmune disease	1 (2.0)	0 (0.0)	0.77
Family history of IBD	9 (18.4)	0 (0.0)	0.17
Family history of colorectal cancer	5 (10.2)	3 (20.0)	0.58
Indication for primary surgical resection			<0.01
Refractory disease	49 (100.0)	0 (0.0)	
Cancer	0 (0.0)	7 (46.7)	
Constipation	0 (0.0)	1 (6.7)	
Diverticulitis	0 (0.0)	5 (33.3)	
Prolapse	0 (0.0)	2 (13.3)	
Anastomotic location			<0.01
Ileal pouch-anal anastomosis (J/S pouch)	24 (48.9)	2 (13.3)	
Ileo-colonic anastomosis	22 (44.9)	1 (6.7)	
Ileo-rectal anastomosis	2 (4.1)	3 (20.0)	
Colo-colonic anastomosis	1 (2.0)	2 (13.3)	
Colorectal anastomosis	0 (0.0)	6 (40.0)	
Recto-anal anastomosis	0 (0.0)	1 (6.7%)	
Pre-procedural medications			
Aminosalicylates	8 (16.3)	0 (0.0)	0.22
Corticosteroids	9 (18.4)	1 (6.7)	0.49
Immunomodulators	15 (30.6)	0 (0.0)	0.04
Biologics	13 (26.5)	0 (0.0)	0.06
Antibiotics	13 (26.5)	1 (6.7)	0.20
Non-steroidal anti-inflammatory drug	1 (2.0)	3 (20.0)	0.04

Value presented as mean ± standard deviation, median (interquartile range), or *n* (%).

IBD, inflammatory bowel disease; ESt, endoscopic stricturotomy.

* Significant comorbidities include congestive heart disease, coronary bypass surgery, chronic obstructive pulmonary disease, renal stone or renal insufficiency, non-gastrointestinal cancer, stroke, and liver failure.

The IBD group included 25 patients with Crohn’s disease (CD) and 24 with ulcerative colitis (UC). The underlying diseases for the non-IBD group that led to bowel resection with anastomosis included cancer (*n *=* *7), diverticulitis (*n *=* *5), prolapse (*n *=* *2), and constipation (*n *=* *1). Regarding anastomotic location, IBD patients consisted of more IPAA (48.9% vs 13.3%, *P *=* *0.01) and ICA (44.9% vs 6.7%, *P *=* *0.01), while non-IBD patients tended to have CRA (40.0% vs 0%, *P *<* *0.01) (Table 1).

IBD patients were on one or more medications for their underlying disease such as: 5-aminosalicylates (*n *=* *8), corticosteroids (*n *=* *9), immunomodulators (*n *=* *15), biologics (*n *=* *13), and antibiotics (*n *=* *13). Non-IBD patients were found to be less likely on relevant medications, as three patients were on NSAIDs, one on corticosteroids, and one on antibiotics (Table 1).

### Characteristics of stricture

The mean age of patients in the IBD and non-IBD groups at the time of diagnosis of anastomotic stricture was 40.2 ± 13.9 and 57.6 ± 11.1 years, respectively (*P *<* *0.01). Most patients were symptomatic at the time of diagnosis (82.9% vs 66.7%, *P *=* *0.27) (Table 2). All the five asymptomatic patients in the non-IBD group had ostomies from previous surgery and had a radiological evaluation in preparation for the closure of ileostomy/colostomy. Gastrografin enemas have been routinely performed to rule out anastomotic leaks or strictures before stoma closure. The strictures were observed on imaging and therefore referred to endoscopic evaluation and intervention before ileostomy closure, while, in the asymptomatic patients of the IBD group, their strictures were incidentally encountered in their routine diagnostic, disease-monitoring, or surveillance endoscopy.

**Table 2.** Characteristics of the strictures and treatments

Characteristic	IBD patients (*n* = 49)	Non-IBD patients (*n* = 15)	*P*-value
Age at diagnosis for strictures, years	40.2 ± 13.9	57.6 ± 11.1	<0.01
Duration from surgery to diagnosis of strictures, years	4.0 (1.1–15.0)	0.5 (0.2–1.0)	<0.01
Age at initial ESt, years	41.7 ± 14.0	57.8 ± 11.0	<0.01
Duration from surgery to initial ESt, years	7.6 (4.1–20.5)	0.6 (0.3–1.0)	<0.01
Symptomatic patients at the time of diagnosis for strictures	34 (82.9)	10 (66.7)	0.84
Diarrhea or urgency	24 (52.2)	6 (40.0)	0.54
Constipation	5 (10.9)	4 (26.7)	0.24
Abdominal pain	21 (45.7)	5 (33.3)	0.51
Nausea and vomiting	6 (13.0)	2 (13.3)	1.00
Multiple strictures	10 (20.4)	1 (6.7)	0.40
Non-traversable strictures	27 (55.1)	12 (80.0)	0.08
Length of the strictures, cm	1.8 ± 0.8	2.5 ± 0.9	0.01
EBD prior to inception ESt	25 (51.0)	1 (6.7)	<0.01
Additional session of ESt	24 (49.0)	6 (40.0)	0.54
Additional session of EBD	10 (20.4)	3 (20.0)	1.00
Total number of ESt			0.57
1 session	25 (51.0)	9 (60.0)	
2 sessions	11 (22.4)	3 (20.0)	
3 sessions	7 (14.3)	1 (6.7)	
4 sessions	2 (4.1)	1 (6.7)	
≥5 sessions	4 (8.2)	1 (6.7)	
Total number of EBD			0.01
0 session	22 (44.9)	12 (80.0)	
1 session	5 (10.2)	1 (6.7)	
2 sessions	7 (14.3)	1 (6.7)	
3 sessions	4 (8.2)	1 (6.7)	
4 sessions	3 (6.1)	0 (0.0)	
≥5 sessions	8 (16.3)	0 (0.0)	
Total session of combined ESt and EBD combined			0.41
0 session	44 (89.8)	12 (80.0)	
1 session	1 (2.0)	3 (20.0)	
2 sessions	1 (2.0)	0 (0.0)	
3 sessions	1 (2.0)	0 (0.0)	
4 sessions	1 (2.0)	0 (0.0)	
≥5 sessions	1 (2.0)	0 (0.0)	
Disease-related hospitalization	10 (20.4)	3 (20.0)	1.00
Disease-related emergency-department visits	7 (14.3)	3 (20.0)	0.90

IBD, inflammatory bowel disease; ESt, endoscopic stricturotomy; EBD, endoscopic balloon dilation.

Multiple strictures were seen in 10 (20.4%) patients in the IBD group and 1 (6.7%) patient in the non-IBD group (*P *=* *0.40). The length of stricture was shorter in IBD patients (1.8 ± 0.8 vs 2.5 ± 0.9 cm, *P *=* *0.01). EBD prior to the inception ESt was documented in 25 (51.0%) and 1 (6.7%) patient in the IBD and non-IBD groups, respectively (*P *<* *0.01) (Table 2).

### Treatment and outcomes

The mean age of the IBD and non-IBD groups at the time of the inception ESt was 41.7 ± 14.0 and 57.8 ± 11.0 years, respectively (*P *<* *0.01). Immediate technical success was achieved in all patients in both groups. Symptomatic improvement after the procedure was reported in 23 patients with IBD (67.6%) and 3 patients with non-IBD (30.0%) (*P *=* *0.06; Table 3). After a median follow-up of 11.0 months (IQR: 3.6–19.0) and 12.3 months (IQR: 3.1–19.5) in the IBD and non-IBD groups, respectively, a median of one (IQR: 1–3) and one (IQR: 1–2) session of the procedures was given to patients in each group. The interval of procedures was numerically shorter in IBD patients than in non-IBD patients (2.3 months [IQR: 1.1–15.1] vs 6.3 months [IQR: 3.8–13.1], *P *=* *0.15). Endoscopic improvement was documented in 10 (20.4%) IBD patients and 5 (33.3%) non-IBD patients (*P* = 0.32; Table 3). Hospitalizations and emergency department (ED) visits due to disease-related symptoms after the initial ESt were comparable between the two groups. Subsequent surgery at the end of the follow-up was required in six (12.2%) patients in the IBD group and four (26.7%) patients in the non-IBD group (*P *=* *0.23; Table 3).

**Table 3.** Outcomes of endoscopic stricturotomy (ESt)

Outcome	IBD patients (*n* = 49)	Non-IBD patients (*n* = 15)	*P*-value
Median duration of follow-up, months	11.0 (3.6–19.0)	12.3 (3.1–19.5)	0.91
Symptomatic improvements	23/34 (67.6)	3/10 (30.0)	0.06
Endoscopic improvements	10 (20.4)	5 (33.3)	0.32
Disease-related hospitalizations	10 (20.4)	3 (20.0)	1.00
Disease-related emergency-department visits	7 (14.3)	3 (20.0)	0.59
Stricture-related surgery after ESt	6 (12.2)	4 (26.7)	0.23
ESt-related complications			
Perforation	0 (0.0)	0 (0.0)	–
Bleeding complication	5/106 procedures (4.7)	0/27 procedures (0.0)	0.20

**Figure 2.**
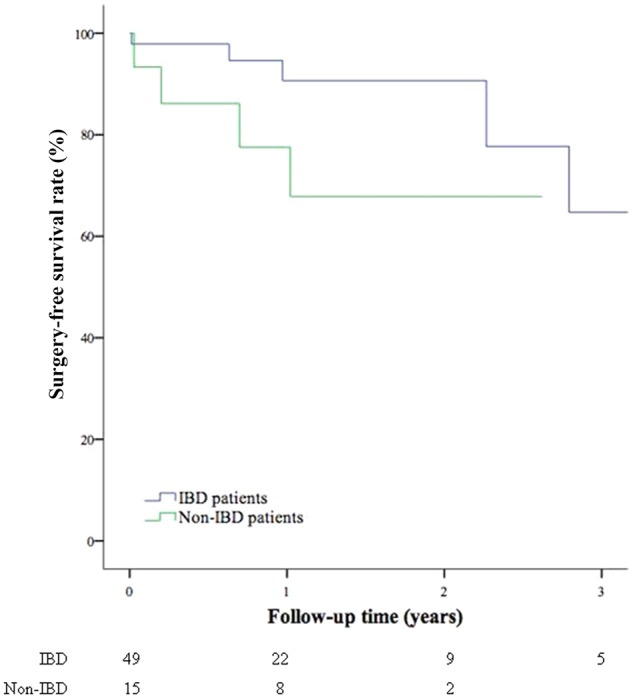
Surgery-free survival curve after being treated with endoscopic stricturotomy in inflammatory bowel disease (IBD) vs non-IBD patients

Kaplan–Meier analysis was conducted to evaluate the surgery-free survival after ESt. IBD patients appeared to have a higher tendency towards remaining surgery-free after the procedure than non-IBD patients (*P *=* *0.08; Figure 2).

### Safety

A total of 106 ESt sessions were performed in 49 IBD patients and 27 ESt sessions were performed in the 15 non-IBD patients. The majority of patients tolerated the procedures well and procedure-associated complication was not common. No perforation was seen in all patients in both groups. IBD patients undergoing ESt tended to have a higher risk of post-procedural bleeding. Blood in the stool was recorded in five occasions (4.7% per procedure) in IBD patients and none in non-IBD patients (*P* = 0.20; Table 3). Three (2.8%) occasions with bleeding after ESt required blood transfusion and the remaining cases resolved spontaneously.

## Discussion

In this historical cohort, we evaluated 64 patients with benign anastomotic strictures treated with ESt. Patients in the IBD group were younger at diagnosis of their primary disease, at surgical resection and stricture diagnosis, as well as at the inception ESt procedure. A majority of strictures in IBD patients were located at the ICA for CD patients or pouch-anal anastomosis site for UC patients, while the most common anastomotic strictures in non-IBD patients were at the CRA, due to underlying disease. Immediate technical success was achieved in all patients in both groups after the initial ESt session and symptomatic improvement was seen more in IBD patients. After about a year of follow-up time, the rates of endoscopic improvement and disease-related hospitalizations or ED visits were similar between the two groups. Subsequent surgical resection of the stricture was needed in twice as many patients in the non-IBD group than those in the IBD group, although the difference was not statistically significant. Nevertheless, the overall surgery-free survival seems to be better in IBD patients. However, the bleeding complication was more prevalent in IBD patients, probably due to the presence of an underlying inflammatory process.

Anastomotic stricture requires mechanical therapy such as EBD, strictureplasty, and surgical resection with anastomosis. While surgery has been the standard therapy for the treatment for most strictures, post-operative complications, as well as a post-operative recurrence of stricture, have been well recognized [15]. As a result, endoscopic approaches have emerged as valid treatment options, with the main goal of the therapy being avoidance or spacing out the need for subsequent surgery. Currently, EBD remains the mainstay in the treatment of both IBD and non-IBD strictures [1, 11, 16–21]. However, it was reported that more than half of CD patients required more than one session of EBD and 66 (35.7%) would eventually need subsequent salvage surgery, during a short period of follow-up [22]. The reported EBD-associated perforation rate was 2%–3% in one review of 24 studies [23]. Apart from treating CD strictures, EBD has proven its efficacy in the treatment of ileal-pouch strictures with a 25-year pouch-retention rate of 85.9% and a complication rate of 2.0% [10, 11]. In a previous study, we also confirmed that the efficacy and safety of EBD in CD patients were similar to those in non-CD patients [1]. In that study, 25/30 (83.3%) non-IBD patients remained surgery-free after EBD and the result is comparable to that reported in the largest series of 94 patients in the literature (89.4%) [21]. In addition, no complication was encountered in non-IBD patients after EBD in that cohort, making the complication rate surprisingly lower than that in the literature (5.3%) [21]. Nevertheless, EBD seems to be a reasonable alternative for both IBD and non-IBD patients for spacing out the need for surgical intervention.

ESt, or previously termed NKSt, has been used for the treatment of various strictures from the biliary system to upper and lower GI [24–26]. ESt in IBD stricture with a needle knife was first reported in our previous study in which the treatment was used for long fibrotic strictures of the ileal pouch refractory to multiple EBD [10]. Subsequently, we reported three cases in which we used NKSt in treating nipple-valve stenosis of the continent ileostomy [27], outlet stricture of the diverted colon [28], and IRA stricture [29]. In our latest study, we confirmed that NKSt was feasible in treating various forms of strictures in patients with IBD with a subsequent surgery rate of 15.3% and a perforation rate of 0.4% [8]. In our cohort comparing EBD and ESt in the treatment of anastomotic CD stricture, we concluded that ESt seems to have a lower rate for subsequent surgery and for perforation than EBD [8]. In comparison with patients who underwent surgery for ICA strictures, ESt also seems to be able to achieve a similar efficacy with a lower complication rate [30]. Although ESt had been reported in our previous study to be used together with EBD in the treatment of non-CD strictures, the outcome of this subgroup of patients was not separately reported in the previous study [1].

The current study is a natural extension of our recent studies, focusing on the treatment of anastomotic stricture in non-IBD patients in comparison to IBD patients. To our knowledge, this is the first case series to evaluate the efficacy and safety of ESt in the treatment of lower GI benign anastomotic strictures in non-IBD patients. Immediate technical success was achieved in all patients in both groups. However, symptomatic improvement was numerically seen more in IBD patients than in non-IBD patients. In this cohort, subsequent surgery was needed in 12.2% of IBD patients, which concurred with what we previously published at 15.3% [8]. However, the need for surgery tended to be higher in non-IBD patients. The reported perforation rate for ESt was 0.4% in our previous cohort [8], but no perforation was seen in patients from both groups in the current study. On the other hand, bleeding complication was seen in 4.7% of occasions in IBD patients in our current study, which concurred the bleeding complication rate reported in the previous cohort (3.9%) [8]. As for non-IBD patients, no bleeding was seen in all patients. The reduction of perforation and bleeding complication rates was possibly promoted by improvement in the ESt technique through exploring different knives to use and also changing the way we cut. Nevertheless, IBD patients had a higher post-procedural bleeding rate, though the difference between the two groups was insignificant. However, we do suspect the increase in bleeding could be due to possible underlying inflammation seen in the IBD patients but not in the non-IBD patients.

There are several clinical implications of this study. It has been identified that ESt was effective in treating IBD anastomotic stricture with a lower rate for subsequent surgery and perforation than traditional EBD. On top of that, it can achieve similar surgery-free survival and recurrence-free survival with surgical resection at a lower rate of morbidity [13]. However, the use of ESt has not been widely adopted on non-IBD strictures. These non-IBD benign anastomotic strictures were also traditionally treated with either EBD or surgery. Though the efficacy of EBD has been proven in many studies in non-IBD anastomotic strictures, the concern for endoscopic perforation has been undoubted. In this cohort, we can see that the overall performance of ESt was comparable in both IBD and non-IBD patients. Although the need for surgery was slightly higher in non-IBD patients numerically, the perforation and bleeding complication rates were much lower in these patients as well. Nonetheless, patients undergoing ESt should be monitored closely. It should be pointed out that the best treatment option should be individualized. The endoscopist should always anticipate perforation or bleeding and be ready when it occurs. The backup plan, such as endoscopic clipping or surgical intervention, should be readily available whenever needed. We recommend that, for patients with a straight stricture of <4 cm, ESt might be a valid alternative before surgery.

There are limitations to this study. Despite being the largest case series of ESt in non-IBD patients and the first series to compare the efficacy of the ESt treatment between IBD and non-IBD patients, the sample size was small and the follow-up time was short. There might have also been a referral and selection bias, as our *i*-IBD Unit is subspecialized in managing complex cases with skilled endoscopists and supporting personnel. This study was also a non-randomized, historical cohort study, the decision on undergoing ESt vs EBD was at the discretion of the treating physician, and a majority of the patients treated with ESt had been previously refractory to EBD therapy. Lastly, the techniques of ESt being a novel procedure remain to be optimized. Therefore, randomized–controlled trials with a larger sample size and a longer follow-up are needed to verify the findings.

In conclusion, our study showed that ESt is a safe and effective way in the management of benign anastomotic strictures in both IBD and non-IBD patients. However, non-IBD patients seem to have a higher need for subsequent surgery but a lower complication rate than IBD patients. The proper use of this technique may help to prolong or avoid the need for stricture-related surgical intervention.

## Authors’ contributions

Study design: L.Z., N.L., and B.S.; data acquisition and analysis: L.Z. and N.L.; manuscript drafting: L.Z. and B.S.; critical review of the manuscript: L.Z., N.L., and B.S. All authors read and approved the final version.

## Supplementary Material

goz051_Supplementary_Video
